# Comparative assessment of oral placement therapy combining B-ARM chewing tube and speech therapy for children with autism spectrum disorders

**DOI:** 10.6026/973206300212040

**Published:** 2025-07-31

**Authors:** Sujata Datta, Ashjan Ashraf Batha, Deepti Jawa, Apurva Chadha, Shipra Jaidka, Madhuri Gupta

**Affiliations:** 1 Department of Paediatric and Preventive Dentistry, Divya Jyoti College of Dental Science and Research, Modinagar, Uttar Pradesh 201204, India

**Keywords:** Autism spectrum disorders, B-ARM, drooling, oral placement therapy, surface electromyography

## Abstract

The impact of oral placement therapy using B-ARM chewing tube along with speech therapy in terms of muscle tonicity and drooling in children with Autism spectrum disorders (ASD) is of interest. 30 children with ASD in the age group of 5-10 years were selected for the study. They were divided into two equal group with speech therapy B-ARM chewy tube provided to experimental group (N=15), while the control group (N=15) was given only speech therapy. The muscle tonicity and drooling of saliva were assessed for both groups using surface electromyography and the drooling severity scale, respectively before and after 3 months. Thus, B-ARM was recommended as an effective tool for oral placement therapy for children with ASD.

## Background:

Autism Spectrum Disorder (ASD) is a multifaceted neurodevelopmental condition characterized by persistent deficits in social communication, restricted interests and repetitive behaviors. In addition to these core symptoms, children with ASD often exhibit a range of physical and neurological challenges, including hypotonia or low muscle tone [1]. Muscle tone plays a vital role in motor development and oral-motor functions such as speaking, chewing, and swallowing [2]. Hypotonia in ASD children can therefore significantly affect their ability to perform everyday tasks and participate in typical communication processes. Recent statistics reveal that approximately 1 in every 44 children globally is diagnosed with ASD [3]. The prevalence of ASD has increased markedly over the past two decades, with data from the Autism and Developmental Disabilities Monitoring (ADDM) Network showing a rise from 1 in 150 children in 2006 to 1 in 54 in 2016 [4]. Genetic predisposition plays a significant role, as evidenced by studies on monozygotic twins, where the concordance rate for ASD can be as high as 90%. In contrast, dizygotic twins show a much lower concordance rate of 2-3%, highlighting the predominance of genetic over environmental influences [5]. Among the less-explored but clinically relevant aspects of ASD is the presence of neuromuscular junction alterations, which may underlie the hypotonia commonly, observed in affected individuals [6]. According to Mei and colleagues, neuromuscular differences may be a key pathological mechanism contributing to the motor delays and low muscle tone often seen in autism [7]. Conventional speech therapy, while effective in enhancing communication skills, may not adequately address the underlying hypotonic conditions. This gap has led to the growing interest in Oral Placement Therapy (OPT), a therapeutic approach that combines tactile, auditory, and proprioceptive inputs to improve muscle tone and oral-motor coordination [8]. OPT focuses on helping individuals "feel" the correct placement and movement of oral structures to facilitate effective speech and feeding behaviors. Unlike traditional speech therapy, which relies heavily on auditory and visual cues, OPT adds a crucial sensory dimension that can be especially beneficial for children with Autism spectrum disorders (ASD), who often have deficits in sensory integration. TalkTools^TM^ defines OPT as "an important addition to traditional speech treatment methods for clients with placement and movement deficits" [9]. Therefore, it is of interest to evaluate and compare the effectiveness of Oral Placement Therapy using the B-ARM chewy tube, in conjunction with speech therapy, in terms of muscle tonicity and drooling in children with ASD using Surface electromyography (sEMG) and the Thomas-Stonell and Greenberg Drooling Rating Scale, respectively.

## Materials and Methods:

This *in vivo*, randomized clinical trial was conducted in the Department of Pediatric and Preventive Dentistry at D.J. College of Dental Sciences & Research, Modinagar, in collaboration with Sparsh Special School and Day Care, Shastri Nagar, Ghaziabad. The study was approved by the Institutional Review Board's Ethical Committee (DJC/IEC/53/2024), and written informed consent was obtained from the legal guardians or caretakers of all participating children after a thorough explanation of the procedures.

## Study population:

A total of 30 children diagnosed with ASD were recruited for the study based on predefined inclusion and exclusion criteria.

## Inclusion criteria:

[1] Children aged 5 to 10 years

[2] Written informed consent provided by parents or legal guardians

## Exclusion criteria:

[1] Presence of other syndromic or neurological conditions

[2] Withdrawal from the study before completion

## Study design:

[1] Participants were randomly divided into two equal groups (n=15 each):

[2] Control Group: Received only conventional speech therapy.

[3] Experimental Group: Received speech therapy along with Oral Placement Therapy (OPT) using the B-ARM chewy tube.

Before the study began, caregivers and children in the experimental group were given a live demonstration of how to use the B-ARM chewy tube. They were instructed to use the device regularly for 3 months, 20 repetitions per day, each lasting approximately 20 seconds.

## Assessment parameters:

## Muscle tonicity:

Muscle tonicity was divided into 3 grades based on the number of muscles involved. In Grade 1, one targeted muscle is involved, in Grade 2, two targeted muscles involvement and in Grade 3, all three targeted muscles are involved. Muscle tonicity was evaluated using surface electromyography (sEMG) [10]. For evaluation of muscle tonicity, the electrodes of surface electromyography were placed on all three targeted muscles, viz. Masseter, Temporalis, and Orbicularis oris muscle. When the targeted muscle was the Masseter muscle, children were instructed to clench their teeth. During the Temporalis muscle, children were asked to perform wide mouth opening, and for the Orbicularis oris muscle, children were instructed to pout. Surface electromyography (sEMG) readings were recorded in real time via connected software and analyzed in graphical format to assess changes in muscle activation levels pre- and post-intervention.

## Drooling severity:

The severity of drooling was assessed using the Thomas-Stonell and Greenberg Drooling Rating Scale, which categorizes the severity of drooling behavior. Thomas-Stonell and Greenberg Drooling Rating Scale 10: 1-Dry (Never drools), 2-Mild (Wet lips only), 3-Moderate (Wet lips and chin), 4-Severe (Wet clothes), 6-Profuse (Wet clothing, hand, trays, objects within reach).

## Results:

Using the statistical package SPSS Version 23, the statistical analysis was conducted. The descriptive statistics included frequency and percentage. The ordinal variables were compared using the Chi-square test, and correlations were established using the chi-squared test. In the control group where B-ARM was not used, the generalized muscle tonicity was degraded further after 3 months, which can be seen as the number of grade 3 and grade 2 muscle involvements in pre-intervention was 73.3% and 26.7%, and post-intervention, it became 80.0% and 20.0%, respectively. But the result was not statistically significant. But in the experimental group where B-ARM was used the generalized muscle tonicity has been improved after 3 month which can be seen as the number of grade 3 and grade 2 muscle in pre intervention group was 80,0% and 20.0% and post intervention it become 6.7% and 73.3% respectively along with 20.0% grade-1 muscle interference. In the experimental group, there was a significant improvement in the muscle tonicity from pre-intervention to post-intervention levels (Table 1 - see PDF). When drooling was evaluated in the control group pre-intervention, 53.3% of children had a score of 1, and 46.7% of children had a score of 2. In the post-intervention phase, 66.7% of children had a score of 1, and 33.3% had a score of 2. In the experimental group pre-intervention, 46.7% had a score of 1, 46.7% had a score of 2, and 6.6% had a score of 3. In the post-intervention, 80.0% had a score of 1, and 20.0% had a score of 2. However, the results were statistically non-significant in both groups (Table 2 - see PDF).

## Discussion:

Oral Placement Therapy (OPT), introduced by Sara Rosenfeld-Johnson in the 1990s, aims to bridge this gap by combining tactile-proprioceptive feedback with traditional techniques, allowing children to better "feel" the correct movements required for effective speech and oral function [11, 12]. Many tools were used for oral placement therapy, Bite Blocks, Z-Vibe, ARK Grabber, TalkTools Horn Hierarchy, TalkTools Straw Hierarchy, Tongue Depressors, Toothette, Nuk Brush, Lip Blocks, Tongue Lifter, Oral Motor Probe, Bite-n-Chew Tip, Bubble Blower, Jaw Grading Bite Blocks, Chewy tube, *etc.* [13]. B-ARM chewy tube has been used in the study because of its easy handling, and is made from non-toxic FDA-approved materials. It is resilient but soft enough for repetitive chewing, it does not collapse or pose a choking risk, and easy for small hands to hold. The "T" or "B Arm" shape gives children a stable grip and promotes independent use during therapy or free time. In individuals with ASD, the masseter, temporalis, and orbicularis oris muscles-key muscles involved in chewing, speaking and facial expression-can often present with atypical muscle tone, impacting oral-motor function [14]. In the present study, the effect of incorporating the B-ARM chewy tube-a textured oral motor tool-into the therapeutic routine of children with ASD was examined. The experimental group, which received OPT alongside speech therapy, showed statistically significant improvements in muscle tonicity as assessed by surface electromyography (sEMG), compared to the control group, which received speech therapy alone. The B-ARM chewing tube combines oral motor stimulation with ball-assisted rhythmic movement to support muscle tonicity in children with ASD. B-ARM regulates tone by providing deep proprioceptive input to normalize muscle tone (especially in hypotonia). This finding supports earlier reports by Ming, Brimacombe, and Wagner (2007), which observed a high prevalence of low muscle tone in autistic children and highlighted the need for targeted motor interventions [15]. The observed improvement in muscle tone aligns with Karen Massey's findings, which emphasized the role of OPT in enhancing speech clarity by addressing underlying muscle weakness and coordination deficits [16]. In the present study, while the control group showed no significant intra-group change in muscle tone after three months, the intervention group demonstrated a marked shift from Grade 3 (involvement of all targeted muscles) to predominantly Grade 2 and Grade 1 classifications. This suggests that regular use of the B-ARM chewy tube contributed to strengthening the orofacial muscles through repetitive and targeted proprioceptive stimulation. When evaluating drooling, however, the results were less definitive. Both groups showed slight improvements over the three months, but these changes were statistically non-significant. This echoes findings by Savitha Sathyaprasad and Annie Elizabeth Abraham, who concluded that while oral motor exercises can help manage drooling, the outcomes may vary based on individual responsiveness, duration of therapy, and the severity of the condition [17]. Despite the lack of statistical significance, it is noteworthy that the intervention group showed a greater proportion of children transitioning to a lower drooling severity score.

## Conclusion:

The B-ARM chewy tube, with its user-friendly design and sensory features, proved to be a practical and well-tolerated tool for young children with ASD. B-ARM chewy tube enhances sensory feedback, enabling clients to develop proper oral placement through repetitive, guided exercises. Its regular use may facilitate the neuromuscular re-education of oral structures required for speech and feeding, especially in cases where traditional therapy alone yields limited gains.
